# Spatial proteomics landscape and immune signature analysis of renal sample of lupus nephritis based on laser-captured microsection

**DOI:** 10.1007/s00011-023-01767-3

**Published:** 2023-07-20

**Authors:** Fengping Zheng, Donge Tang, Shanshan Li, Zhifeng Luo, Yueqi Song, Yinxin Huang, Qing Gan, Hanyong Liu, Xinzhou Zhang, Dongzhou Liu, Qingwen Wang, Zuying Xiong, Yong Dai

**Affiliations:** 1 Department of Nephrology, Peking University Shenzhen Hospital, Shenzhen Peking University, The Hong Kong University of Science and Technology Medical Center, Shenzhen, 518036 Guangdong China; 2 The Second Clinical Medical College, Shenzhen People’s Hospital, Jinan University, Shenzhen, 518020 Guangdong China; 3 The Second Department of Urology, Affiliated Hospital of Guilin Medical University, Guangxi, 541001 China; 4 Department of Rheumatism and Immunology, Peking University Shenzhen Hospital, Shenzhen, 5218036 Guangdong China; 5 School of Medicine, The First Affiliated Hospital, Anhui University of Science and Technology, Huainan, 232001 Anhui China

**Keywords:** Lupus nephritis, Glomerulus, Interstitial, Tubules, Immune

## Abstract

**Objective:**

We aimed to reveal a spatial proteomic and immune signature of kidney function regions in lupus nephritis (LN).

**Material and methods:**

The laser capture microdissection (LCM) was used to isolate the glomerulus, tubules, and interstitial of the kidney from paraffin samples. The data-independent acquisition (DIA) method was used to collect proteomics data. The bioinformatic analysis was performed.

**Results:**

A total of 49,658 peptides and 4056 proteins were quantitated. Our results first showed that a high proportion of activated NK cells, naive B cells, and neutrophils in the glomerulus, activated NK cells in interstitial, and resting NK cells were accumulated in tubules in LN. The immune-related function analysis of differential expression proteins in different regions indicated that the glomerulus and interstitial were major sites of immune disturbance and regulation connected with immune response activation. Furthermore, we identified 7, 8, and 9 hub genes in LN’s glomerulus, renal interstitial, and tubules. These hub genes were significantly correlated with the infiltration of immune cell subsets. We screened out ALB, CTSB, LCN2, A2M, CDC42, VIM, LTF, and CD14, which show higher performance as candidate biomarkers after correlation analysis with clinical indexes. The function within three regions of the kidney was analyzed. The differential expression proteins (DEGs) between interstitial and glomerulus were significantly enriched in the immune-related biological processes, and myeloid leukocyte-mediated immunity and cellular response to hormone stimulus. The DEGs between tubules and glomerulus were significantly enriched in cell activation and leukocyte-mediated immunity. While the DEGs between tubules and interstitial were enriched in response to lipid, antigen processing, and presentation of peptide antigen response to oxygen-containing compound, the results indicated a different function within kidney regions.

**Conclusions:**

Collectively, we revealed spatial proteomics and immune signature of LN kidney regions by combined using LCM and DIA.

**Supplementary Information:**

The online version contains supplementary material available at 10.1007/s00011-023-01767-3.

## Introduction

LN is a severe complication of SLE, but it is the most common complication. At least 50% of SLE patients will develop LN, and 10–20% will progress into end-stage renal disease (ESRD) within 5 years [1, 2]. Despite the application of immunosuppressive therapy, the incidence of LN remained at a high level, and the treatment of LN is difficult to make a milestone to improve the prognosis, probably caused by the complex immune microenvironment of LN [3]. Abnormalities of innate and adaptive immunity were essential components of the pathogenesis of lupus. The production of autoantibodies against nuclear and cellular antigens and renal deposition of immune complexes, as well as activation of dendritic cells and interactions of T and B cells within the kidney, complement activation, and other pathways, can lead to renal inflammation and damage [4]. The disorder of the immune environment of the kidney was critical to the prognosis of the kidney with LN. Therefore, the immune regulatory agents were the promising options for treating lupus nephritis [5].

The generally accepted LN classification system of the International Society of Nephrology and Renal Pathology (ISN/RPS) mainly focused on glomerular pathology—assessment of cellular composition and presence of immune complexes in glomeruli by light and electron microscopy [6], but this was not consistent with the prognosis of patients completely [7–9]. In some cases, interstitial injury, rather than glomerular injury, was more predictive of renal prognosis [9]. Furthermore, the damage degree of interstitial was significantly correlated with poor response to treatment [10]. Interstitial inflammation and tubular atrophy were the significant symptoms of renal prognosis, but they were not necessarily to synchronized. Detailed analysis of the whole pathology of the kidney is rare and challenging. Though high-dimensional cellular analysis might achieve, single-cell RNA sequencing could describe cell populations in small biopsy samples from damaged kidney tissue [11]. However, this method has high requirements for samples, The small kidney biopsy specimens need to be preserved in a way of preserving intact and living cells. The bulk tissue was often used as the research object, in which there is inability to distinguish the same cell type from different compartments. As we know, the paraffin block of the kidney tissue is the most often storage method. Therefore, how to make a full use of these paraffin blocks is also attractive for the researchers and the clinicians.

Proteins are molecules that directly perform functions and can better reflect the functional activities of cells. Studies have shown that the proteomics in urine is highly correlated with the proteomics in the kidney [12], indicating that non-invasively monitoring the immune status in the kidney or determining disease features that can be used for diagnosis. Detailed analysis of the immune profile of distinct compartments within the kidneys of LN patients may provide new insights into pathways associated with histopathology and help delineate disease heterogeneity. Such insights may inform treatment decisions and guide the development of new drugs.

In the present study, we used laser microdissection, which can isolate anatomic tissue compartments, and DIA protein quantification techniques to analyze the immunological characteristics of glomeruli, interstitial, and tubules from renal tissue paraffin sections of LN patients to draw a spatial proteomics landscape and immune signature, which might help us better understand molecular mechanisms underlying different regions of the LN kidney and discover potential diagnostic/prognostic markers.

## Materials and methods

### Patient samples

The human studies involved were approved by the ethics committee of Shenzhen People’s Hospital. Informed consent for human tissues for research purposes was obtained from all patients recruited in this study. 21 LN tissues from LN patients and 11 normal control kidney tissues (NC) collected from para-cancer tissue of renal carcinoma without pathological changes and invasion and metastasis to tubule were included in proteomic sequencing. All the patients were diagnosed with LN according to the 2003 International Society of Nephrology/Renal Pathology Society classification [6]. The LN activity and chronicity were determined according to the National Institutes of Health (NIH) activity (NIH-AI) and chronicity indices (NIH-CI) [13]. The clinical parameters are shown in Table 1.

**Table 1:** The clinical parameters for study population

Group	LN	Control
Age (years)	31.28 ± 13.19	38.5 ± 5.4
Sex (female/male)	17/4	0/11
BUN	10.8 ± 5.67	11.47 ± 2.3
C3 (mg/dl)	0.53 ± 0.38	0.64 ± 0.53
C4 (mg/dl)	0.095 ± 0.09	0.46 ± 0.43
Hemoglobin (g/L)	92.31 ± 19.18	117 ± 23.55
White Blood Cell (10^9^/L)	8.3 ± 4.67	11.09 ± 4.42
Lymphocytes (10^9^/L)	1.7 ± 1.55	1.8
Thrombocyte (10^9^/L)	262.43 ± 108.58	285.85 ± .70
Proteinuria/24 h (g/L)	2.94 ± 1.35	N
Cystatin C (mg/L)	2.42 ± 1.01	2.76 ± 1.31
dsDNA (IU/mL)	468 ± 461	N
SCr (μmol/L)	128.86 ± 50.82	97.14 ± 21.43
IgA (g/L)	2.35 ± 1.14	N
IgG (g/L)	11.9 ± 6.46	N
IgM (g/L)	1.07 ± 0.52	N

### Sample prepared and LCM

For mass spectrometry (MS) data collection in data-dependent acquisition (DDA) mode, six formalin fixation and paraffin-embedding tissue samples (3 from LN and 3 from the normal kidney) were collected from the renal needle puncture and nephrectomy specimens within 1 h after removal. The section size is about 5–7 mm long and 0.5–1 mm wide, with a thickness of 10 μm. To acquire mass spectrometry data in data-independent acquisition (DIA) mode, the paraffin block of patient samples from renal needle biopsy was sectioned into 10 μm-thick with a clean blade and transferred to a thermoplastic membrane slide (PET Frame Slide, steel frames, RNase-free Leica, Germany). The glomerulus, tubules, and interstitial structures were selectively isolated by Leica microsystems LMD System (LMD7000, Leica, Germany) with a UV laser (10 × microscope objective). The characteristic set as power was set for 38, the aperture was 14, and the speed was 4. The tissue fragments dropped into cap tubes due to gravity. 5–8 glomerular, tubules, and interstitial structures of the same size were obtained from each slide. The represented images are shown in Supplementary Fig. 1.

### Protein extraction, enzymolysis, and desalted

Samples were centrifuged at 20,000*g* at room temperature for 10 min. Then the samples were processed by following steps—add 20 μL of 50 mM aqueous ammonium bicarbonate containing dithiothreitol (DTT) concentration of 10 mM; 95 °C metal bath reaction 30 min; iodoacetamide (IAM) was added immediately after the end temperature of the reaction was reduced to room temperature, the final concentration was 50 mM, and it was allowed to stand for 30 min at room temperature protected from light. The sample tube was placed into the water bath sonicate for 20 min. 0.2 μg of trypsin enzyme was added to each sample tube for enzymatic lysis, followed by shaking and mixing well for 30 s, 800*g* centrifugation at room temperature for 10 s, enzymatic digestion in a water bath pot at 37 °C for 14 to 16 h. Activation: take a new C18 column, use 1 mL methanol to pass the column, and flow rate 3 drops/s. Equilibrium: use 1 mL 0.1% FA over the column, flow rate 3 drops/s. Loading: Protein liquid sample diluted with SDS-free (sodium lauryl sulfate) protein lysate to 1 mL over-column flow rate 1 drop/s. Washing: use 1 mL 0.1% FA over the column, flow rate 3 drops/s, and repeat 3 times. Elution: slowly elute with 800uL 75% ACN at a flow rate of 0.5 drops/s, and collect the eluent. Drainage: the eluent was then frozen and drained.

### Data analysis

The experimental procedure of High pH RP separation and DDA and DIA analysis by nano-LC–MS/MS is described in supplementary methods. DDA data were identified using MaxQuant (version 1.5.3.30). Peptide/protein entries that satisfied FDR ≤ 1% were used to build the final spectral library. Data was reviewed according to the UniProtKB/Swiss-Prot *H*. *sapiens* proteome database. Parameters were selected as follows: (1) Enzyme: Trypsin; (2) Minimal peptide length: 7; (3) PSM-level FDR and Protein FDR: 0.01; (4) Fixed modifications: Carbamidomethyl (C); (5) Variable modifications: Oxidation (M); Acetyl (Protein N-term). DIA data was analyzed using Spectronaut (version 12), using iRT peptides to calibrate retention time. FDR was estimated using the mProphet scoring algorithm, which accurately reflects the matching degree of ion pairs. Then, based on the target-decoy model applicable to SWATH-MS, the false positive control is completed with FDR not exceeding 1%. MSstats (quantile normalization) screened DEPs according to a difference multiple ≥ 1.5 and *P* < 0.05 as the criteria for determining significant differences.

### Identification and correlation of disease immune infiltrate cells

To determine the enrichment levels of classical immune signatures, we performed single sample gene set enrichment analysis (ssGSEA) on the log2-transformed, normalized gene expression data using GSVA (v.1.38.2) in each sample using the R package. The infiltration levels of 28 immune cell types were quantified based on 782 genes expressed explicitly in specific immune cell types [14]. The CIBERSORT algorithm, which can perform linear support vector regression to deconvolute gene expression profiles, was used to estimate the number of immune cells in samples [15]. We assessed the proportions of 22 types of infiltrating immune cells in all samples using the CIBERSORT method in R software and showed the immune cell composition of patients with varied immune patterns using immune cell content stacking plot. Differences in immune cell proportions were evaluated using the Wilcoxon rank-sum test.* P* < 0.05 was considered statistically significant.

### Immune-related genes identified

To obtain more effective convinced proteins on behalf of three regions of the kidney in LN and control samples, functional analysis and downstream associations with DEPs were restricted to the 4880 protein groups. The number of “NA” identified by MSSTAT was observed in > 50% of samples (Table S1). 17,731 immune-related genes were obtained from ImmPort databases (https://www.immport.org/). The DEGs with an adjusted *P* value < 0.05 and absolute fold-change > 1.5 were considered significant and employed for further analysis.

### Bioinformatic analyses

The differentially expressed genes between different groups and immune-related genes among them were conducted by Gene ontology (GO) and KEGG enrichment analysis to further elucidate the biological, cytology role, and related pathway. To investigate the differences in biological processes between different subareas, based on a dataset of gene expression profiles from LN and NC samples, we performed gene set enrichment analysis (GSEA). GSEA is commonly used to estimate the changes in the pathways and biological activities in the samples of expression datasets. In the study, the GSEA was performed to functionally elucidate the biological roles of the DEPs in the part of the immune. The gene sets and “c5.go.v7.2.symbols.gmt” were retrieved from the MSigDB database for use in the GSEA. The analysis was based on the cluster Profiler and enrich plot package. *P* values < 0.05 and FDR < 0.05(BH) were considered as statistically significant.

### Protein–Protein Interaction (PPI) Network and hub gene identified

PPI network analysis was constructed with the immune-related DEPs using the STRING interactome (https://string-db.org/). CytoHubba was employed to screen hub genes signatures in the network. Five models including MCC, DMNC, MNC, Degree from CytoHubba, identified the hub genes.

### Statistical analysis

Correlation coefficients between different genes were estimated via Pearson and Spearman correlation analyses. All statistical *P* values were two-sided, and *P* < 0.05 was considered statistically significant.

## Results

### FFPE kidney tissue workflow

**Fig. 1:**
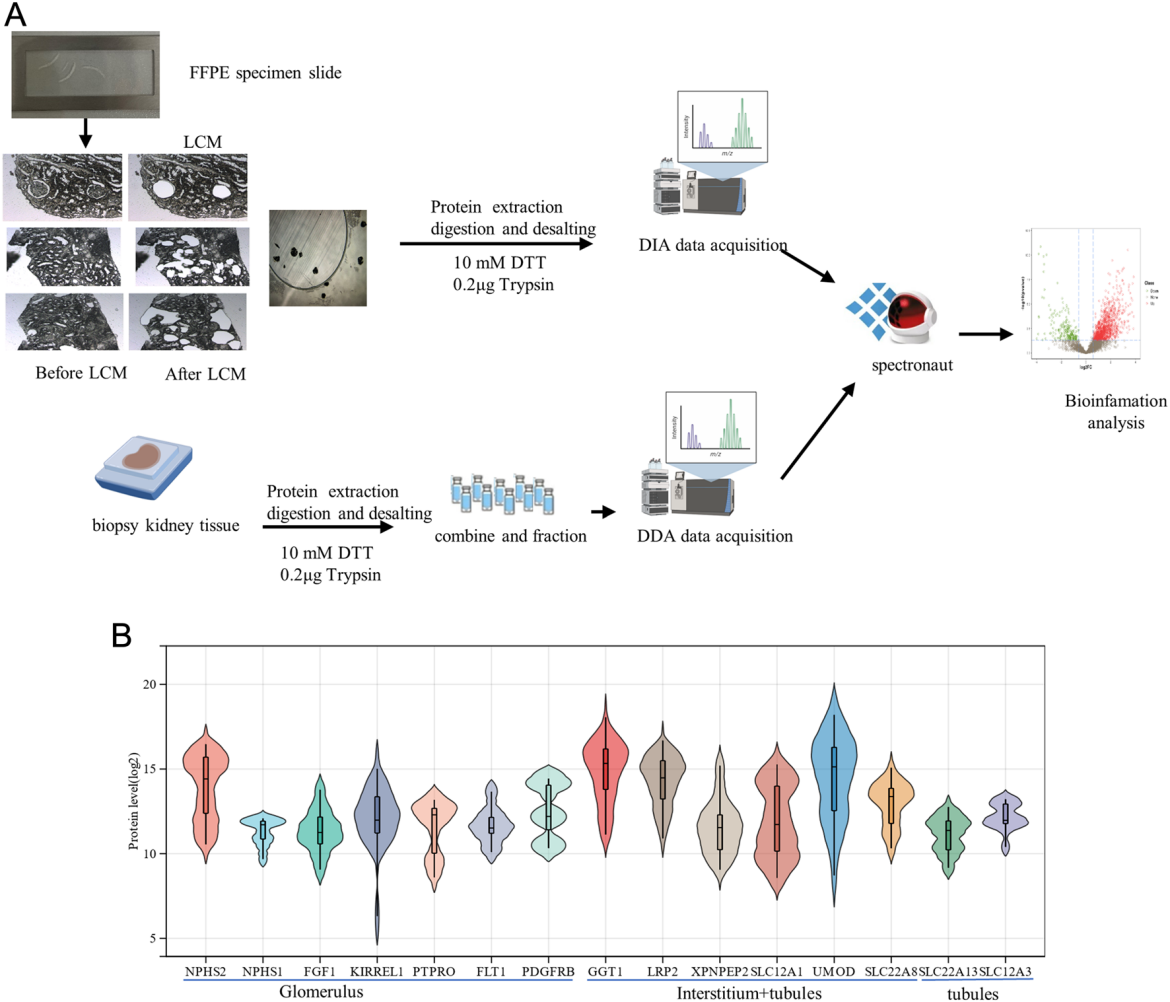
**A** Scheme of the proteomic analysis. **B** The specific proteins expressed in the three renal compartments

One of the highlights of our method permitted the analysis of such a minimal sample from FFPE blocks. The procedure is schematically illustrated in the graphical abstract (Fig. 1A). The different regions of kidney tissue from 10 μm FFPE were isolated using LCM. The proteomic profiles of different regions of kidney tissue were depicted in combination with the technique of DIA protein quantification. The samples of interest went through mass spectrometry data collection in DDA mode. The number of peptides and proteins in the spectral library was 81,374 and 8309, respectively. Our data also identified the kidney-specific proteins in the glomerulus, tubules, and interstitial, respectively (Fig. 1B). Basic statistics of the DDA identification results, including unique peptide distribution, protein mass distribution, and protein coverage distribution, were shown in supplementary Fig. S1A. 95 samples in total contributed mass spectrometry (MS) data in DIA mode. 49,658 peptides and 4056 proteins were quantitated using MSstats software packages. The quantitative statistics of each sample are shown in supplementary Fig. S1B.

### Immune cell infiltration patterns characterized by different regions of the kidney

LN is an immune disease. The alteration of the immune microenvironment in the kidney contributes greatly to the progression of the LN. The CIBERSORT algorithm was used to assess the relative subpopulations of immune cell infiltration, including 22 distinct leukocyte subsets among three regions in the kidney of the LN and control group. The ssGSEA was performed to show the immune signature scores. CIBERSORT analysis showed that patients with LN had a high proportion of activated NK cells, naive B cells, and neutrophils in the glomerulus. These three cell types make up 19.0%,11.8%, and 11.1% of the cell subsets, respectively. In the renal interstitial, the LN group was associated with a high proportion of activated NK cells (14.8%). However, the NC group was positively correlated with T regulatory cells (Tregs) (18.8%). In the renal tubule, resting NK cells accounted for the largest proportion in both LN and NC groups with a proportion of 24.4% and 35.7% (Fig. S2A). These results indicated different immune cell infiltration into different regions of the LN kidney.

The correlation between LN and the immune cells was also calculated (Fig. S2B). The correlation of LN and NC was different, indicating that the disease affected the distribution of immune cells. In the LN glomerulus, plasma cells, naive B cells, naive CD4 T cells, CD8 T cells, eosinophils, and neutrophils showed a significant positive correlation. In contrast, T follicular helper cells and activated T cells CD4 memory showed a significant negative correlation. Similarly, in the renal interstitial, activated NK cells showed a negative correlation with memory B cells but a positive correlation with activated memory T cells. M2 macrophages and neutrophils also showed a positive correlation. In renal tubules, eosinophils and activated mast cells have a positive correlation. T follicular helper cells, M0 macrophages, and naive B cells also correlate positively.

### Different Immune Cell Infiltration between LN and NC group

**Fig. 2:**
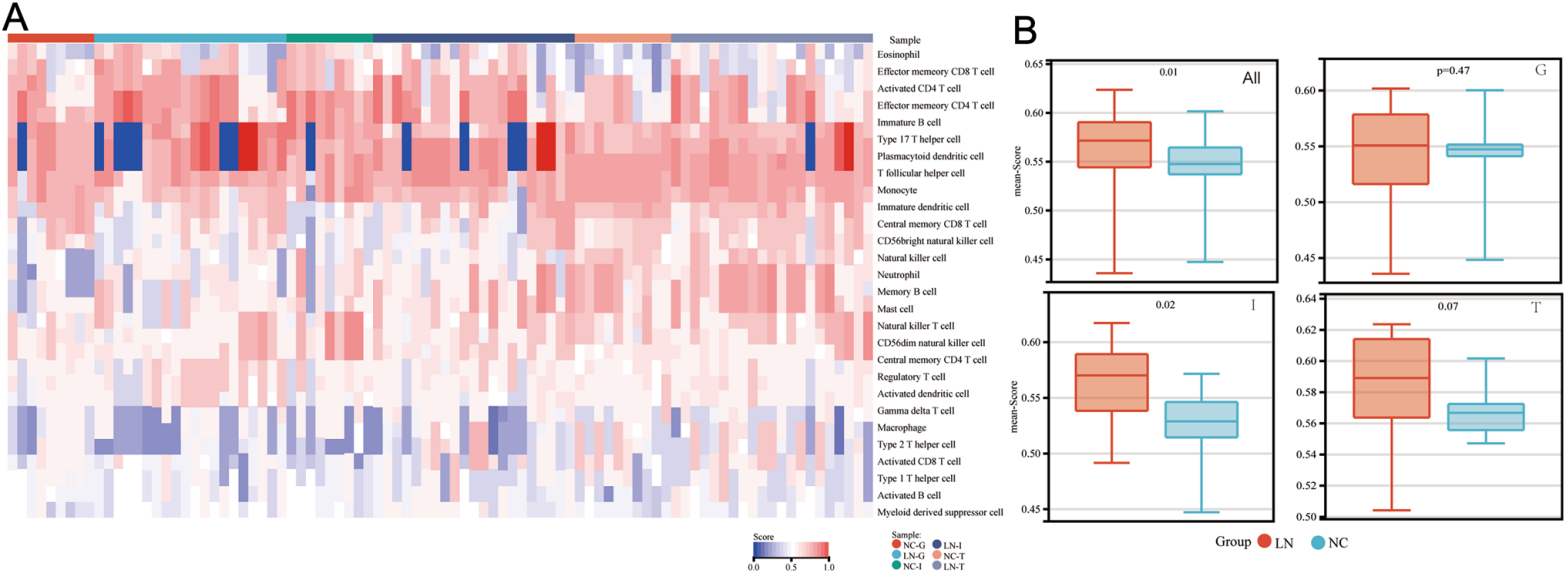
Overview of the immune enrichment score based on ssGSEA analysis. **A** Heatmap of immune enrichment score of three compartments in both LN and NC kidney samples. **B** Boxplot of the comparison of immune enrichment scores between the two groups. *G* glomerulus, *I* interstitial, *T* tubules

To compare the differences in immune infiltration between LN and NC groups, ssGSEA (Fig. 2) and CIBERSORT (Fig. S2) were used. Principal component analysis (PCA) of the enrichment score and proportion of immune cells indicated that the two groups could be well discriminated, especially in renal interstitial (Fig. S3). In addition, the immune signature scores of LN samples were greater than those of NC samples, especially in the renal interstitial samples (*P* < 0.05) (Fig. 2B).

**Fig. 3:**
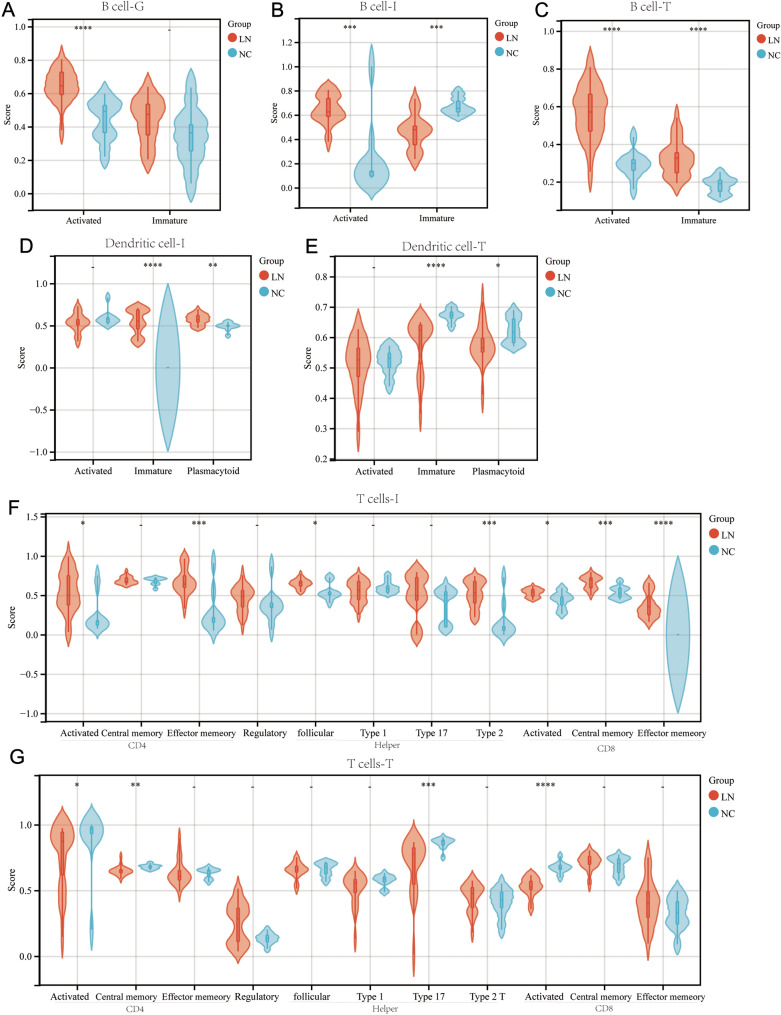
Immune signature Violin diagram between LN and NC group. Enrichment score of B cell in **A** glomerulus, **B** interstitial, **C** tubules. Enrichment score of dendritic cells in **D** interstitial and **E** tubules. Enrichment score of T cell in **F** interstitial and **G** tubules. Red indicates LN kidney samples; green indicates NC kidney samples

Activated B cells in the glomerular of the LN group have higher immune signature scores (Fig. 3A) while the analysis revealed that patients with LN have significantly higher levels of naive B cells, CD8 T cells, activated mast cells, and eosinophils than in NC, representing more adaptive immune cells. However, lower levels of M2 macrophages and activated dendritic cells than in NC are noted (Fig. S4A).ssGSEA analysis showed that activated B cells, immature B cells, and immature and plasmacytoid dendritic cells had higher immune signature scores in renal interstitial samples of the LN group (Fig. 3B, D). Among T cells, the activated and effector memory CD4 T cell, all types of CD8 T cells, including activated CD8 T cell, central memory, and effector memory CD8 T cell had higher immune enrichment scores in the LN group (Fig. 3F). The renal interstitial samples in LN showed a higher proportion of gamma delta T cells and neutrophils. Still, they exhibited a remarkably lower infiltration ratio of resting CD4 memory T cells, follicular helper T cells, regulatory T cells (Tregs), and eosinophils than the NC group (Fig. S4B). These results showed that the renal interstitial had a stronger cellular immune response in the LN group.

In the normal tubule samples, the enrichment level of activated and immature B cells was elevated in the LN group (Fig. 3C). However, interestingly, the enrichment scores of the activated and central memory CD4 T cell, activated CD8 T cell, and Th17 helper cell were reduced in LN group (Fig. 3G), which means lower T cell activation in renal tubules during LN stage, or may be the results of cell migration. The same situation happens with immature and plasmacytoid dendritic cells (Fig. 3E).

In the normal tubule samples, plasma cells, CD8 T cells, follicular helper T cells, gamma delta T cells, resting dendritic cells, and activated mast cells were not identified using the CIBERSORT algorithm. While compared with the LN group, regulatory T cells were significantly higher in renal interstitial and tubules (Fig. S4C), consistent with the fact that regulatory T cells lessen during the SLE progression.

Overall, B cells, plasma, and CD8 + T cells were all elevated in the LN group’s glomeruli, interstitial, and tubules. As the strongly antigen-presenting cells, DC cells significantly increased in the LN group, mainly distributed in renal interstitial and around tubules. Therefore, the LN group showed more active and abundant immune cells than the NC group, leading to a response to disease progression.

### Immune-related functional enrichment analysis of DEPs

**Fig. 4:**
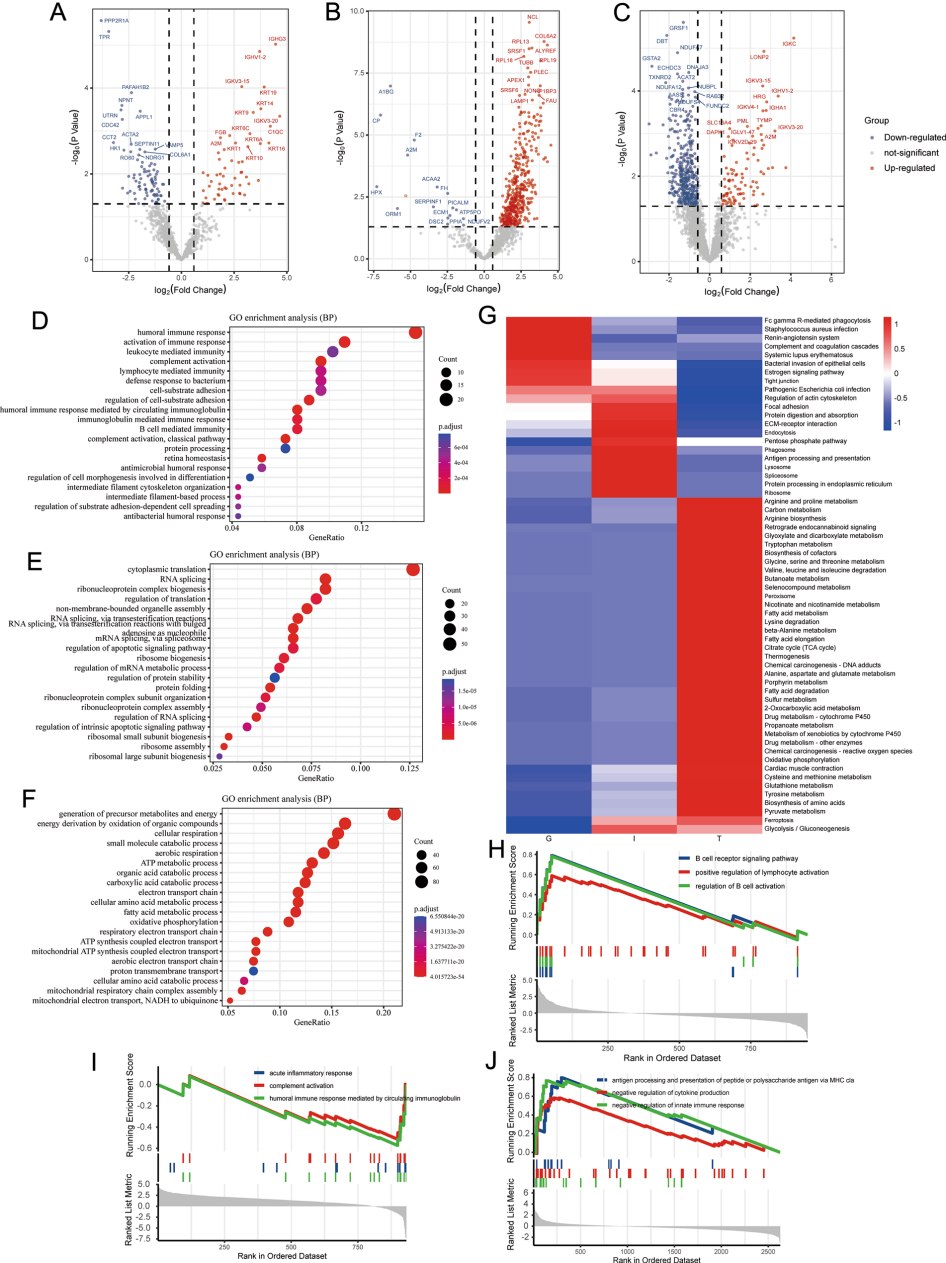
The volcano plots from different kidney compartments, **A** glomerulus, **B** interstitial, **C** tubules. The GO enrichment analysis in DEPs of different kidney compartments, **D** glomerulus, **E** interstitial, **F** tubules. **G** The comparison of KEGG pathways of DEPs from the renal glomerulus, interstitial, and tubules. GSEA enrichment plots of immune-related pathways in each compartment

A differential study of LN samples and NC samples revealed that compared with the NC group, 145 DEPs in the glomerulus (60 of them were upregulated and 85 were downregulated) (Fig. 4A), 443 DEPs in renal interstitial (427 of them were upregulated and 16 were downregulated) (Fig. 4B), 466 DEPs in kidney tubules (99 were upregulated and 367 were downregulated) (Fig. 4C), in LN group. Then, the GO and KEGG analysis was performed. The biological process of GO analysis shows that the DEPs in the glomerulus were mainly involved in immunologic disorder terms, such as humoral immune response, activation of the immune response, complement activation, lymphocyte-mediated immunity, and B cell-mediated immunity (Fig. 4D). The GO terms of renal interstitial include biology associated with organizational structure and translation (Fig. 4E). In contrast, the enriched terms of the biological process were related to metabolic including generation of precursor metabolites and energy, energy derivation by oxidation of organic compounds, cellular respiration, ATP metabolic process, oxidative phosphorylation, and fatty acid metabolic process in tubules (Fig. 4F).

To compare the overall difference between the DEPs with pathway, heat maps of the KEGG pathway of the three parts of the kidney were drawn (Fig. 4G). The significantly enriched KEGG signaling pathways appear to show distinct differences between the three regions. Fc gamma R-mediated phagocytosis, complement and coagulation cascades, *Staphylococcus aureus* infection, Systemic lupus erythematosus, and estrogen signaling pathway were enriched in the glomerulus, which is more relevant to the features of lupus. At the same time, the pathway of antigen processing and presentation and endocytosis were mainly enriched in the renal interstitial. Notably, the DEPs of tubules showed clear metabolic-related biology, and the enrichment of immune-related pathways was not noticeable.

To further analyze the immune aspect difference, we performed GSEA-GO on all genes in three parts between LN and normal samples, respectively. The immune-related biological processes, such as the B cell receptor signaling pathway, regulation of B cell activation, and positive regulation of lymphocyte activation, were activated in the glomerulus (Fig. 4H). In contrast, the biological processes, such as humoral immune response mediated by circulating immunoglobulin, acute inflammatory response, and complement activation, were inhibited in renal interstitial (Fig. 4I). The immune-related biological processes, such as negative regulation of innate immune response, negative regulation of cytokine production, and antigen processing and presentation of peptide or polysaccharide antigen via MHC class II, were activated in tubules (Fig. 4J).

These results suggested that the glomerulus and interstitial were significant immune disturbance and regulation sites.

### Immune-related DEPs analysis

The immune-related key genes might significantly influence LN's clinical outcome. Herein, we retrieved immune-related genes from the ImmPort dataset to predict the function of DEPs.

**Fig. 5:**
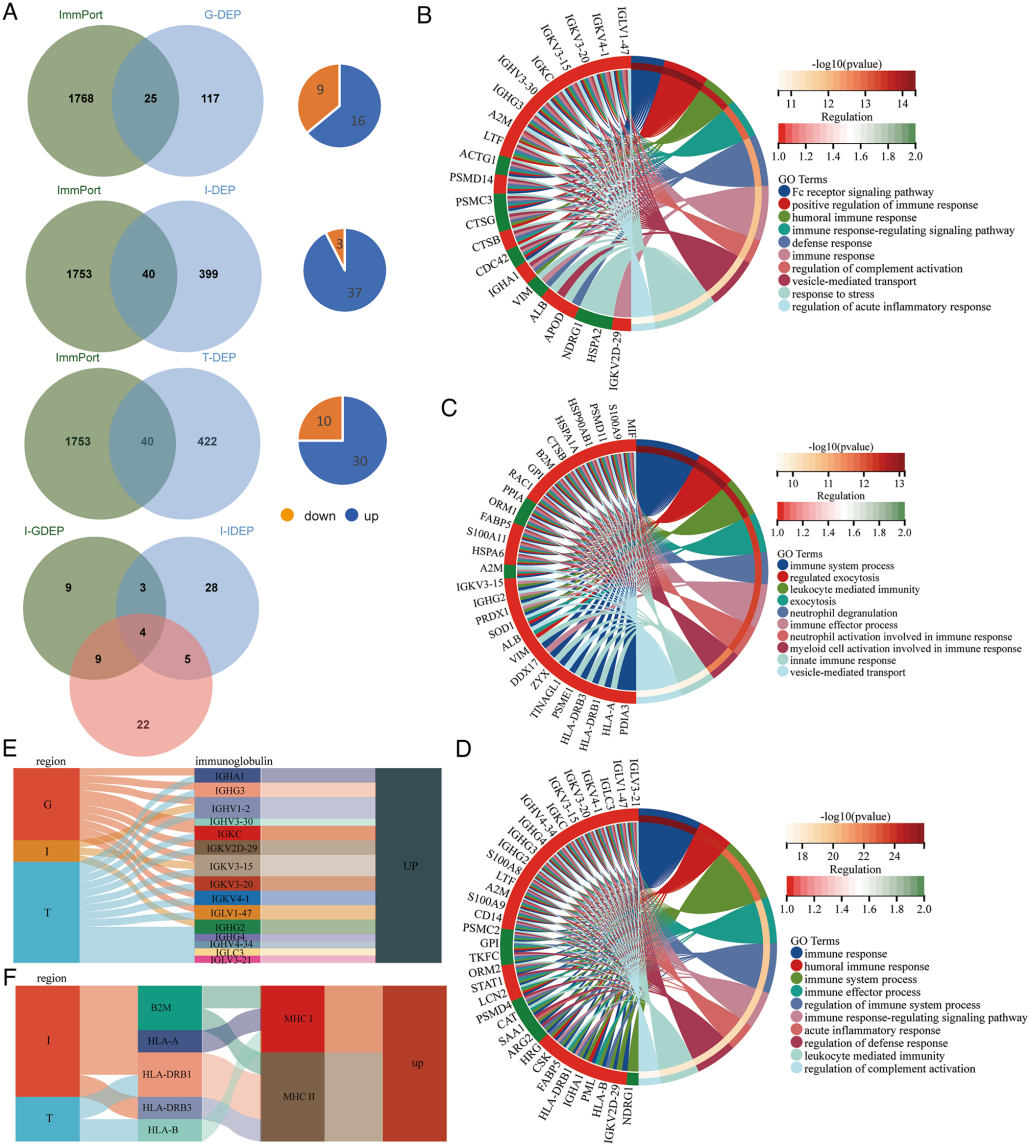
**A** Immune-related gene from ImmPort data sources versus DEPs between LN and NC group from three renal compartments Venn diagram, the pie chart shows the dysregulated of immune-related DEPs. GO enrichment analysis of immune-related DEPs from different compartments, **B** glomerulus, **C** interstitial, **D** tubules. **E** The Sankey diagram shows the expression of immunoglobulins in different compartments. **F** The Sankey diagram shows the expression of MHC I/II molecules in different compartments

The intersections of DEPs in three regions on immune-related genes from the ImmPort dataset are shown in Venn diagrams (Fig. 5A). There were 25, 40, and 40 immune-related DEPs in the glomerulus, interstitial, and tubules, respectively. Furthermore, we found higher expression of immune-related genes in the LN group, which was compatible with the higher infiltration of immune cells. These dysregulated immune-related genes may invoke an immune disorder in the LN. Notably, ALB, A2M, IGHV1-2, and IGKV3-15 were dysregulated in all regions, and ALB, IGHV1-2, and IGKV3-15 upregulated in the LN group, indicating that these genes may play important roles in participating immune regulation in LN.

Subsequently, we annotated these immune-related DEGs at three regions through biological processes in Gene Ontology (Fig. 5B–D). Biological process analysis of the DEGs revealed immune biology in three regions was unlike, which illustrated that different regions of kidney play different roles in the way that they participate in the immune response in LN compared with NC. The LN group expressed higher amounts of immunoglobulin and MHC I- and II-related antigen-presenting molecules than the NC group, resulting in stronger immunogenicity (Fig. 5E, F).

### Correlation between immune-related genes and immune cells

Correlations between immune infiltrate cells and immune-related genes in different regions were performed to analyze the potential status of immune-related genes further.

**Fig. 6:**
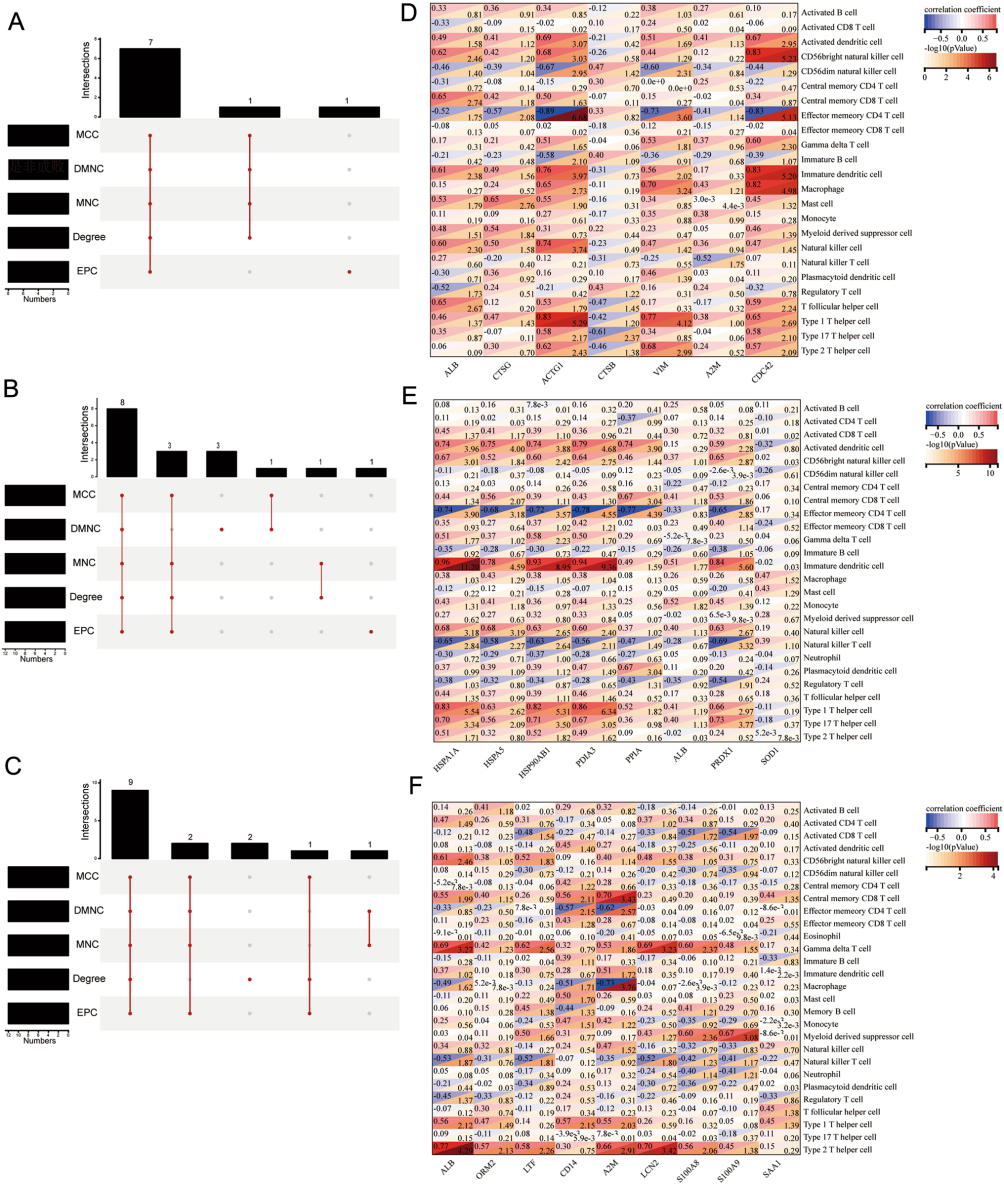
Identification of hub genes. **A** Five algorithms have screened out 7 overlapping hub genes in the glomerulus. **B** Five algorithms have screened out 8 overlapping hub genes in the interstitial. **C** Five algorithms have screened out 9 overlapping hub genes in tubules. **D**–**F** Correlation analysis between hub genes in renal glomerulus, interstitial and tubules and enrichment scores of 28 immune cells type in LN, respectively

To find the key immune-related DEPs that play an important role in the biological process and may play a leading regulatory role in the pathway, protein–protein interaction (PPI) network analysis was performed on immune-related genes using STRING, and 24 hub genes—all ranked in the top five algorithms—were obtained using Cyto-Hubba. There were 7, 8, and 9 hub genes in the glomerulus, renal interstitial, and tubules, respectively (Fig. 6A–C).

To further examine the value of these hub genes, correlation analysis between immune enrichment scores of infiltrate cells and immune-related hub genes in different regions was performed and shown in Fig. 6D–F. Meanwhile, the correlation based on these hub genes and immune infiltrate cells in separate compartments are shown in Fig. S5. Notably, ACTG1, VIM, ALB, CDC42, and CTSB in the glomerulus, HSPA5, HSPAB1, HSPAIA, PDIA3, PPIA, HSP90AB1, PRDX1, and ALB in renal interstitium, and A2M, ALB, LCN2, LTF, CD14, S100A8, and S100A9 in tubule were discovered to be significantly correlated with the enrichment scores of immune cell subsets (*P* < 0.05), significantly different between LN and NC groups (*P* < 0.05). These results suggested that these genes are more likely to play an important role in the immune pathogenesis of LN.

**Fig. 7:**
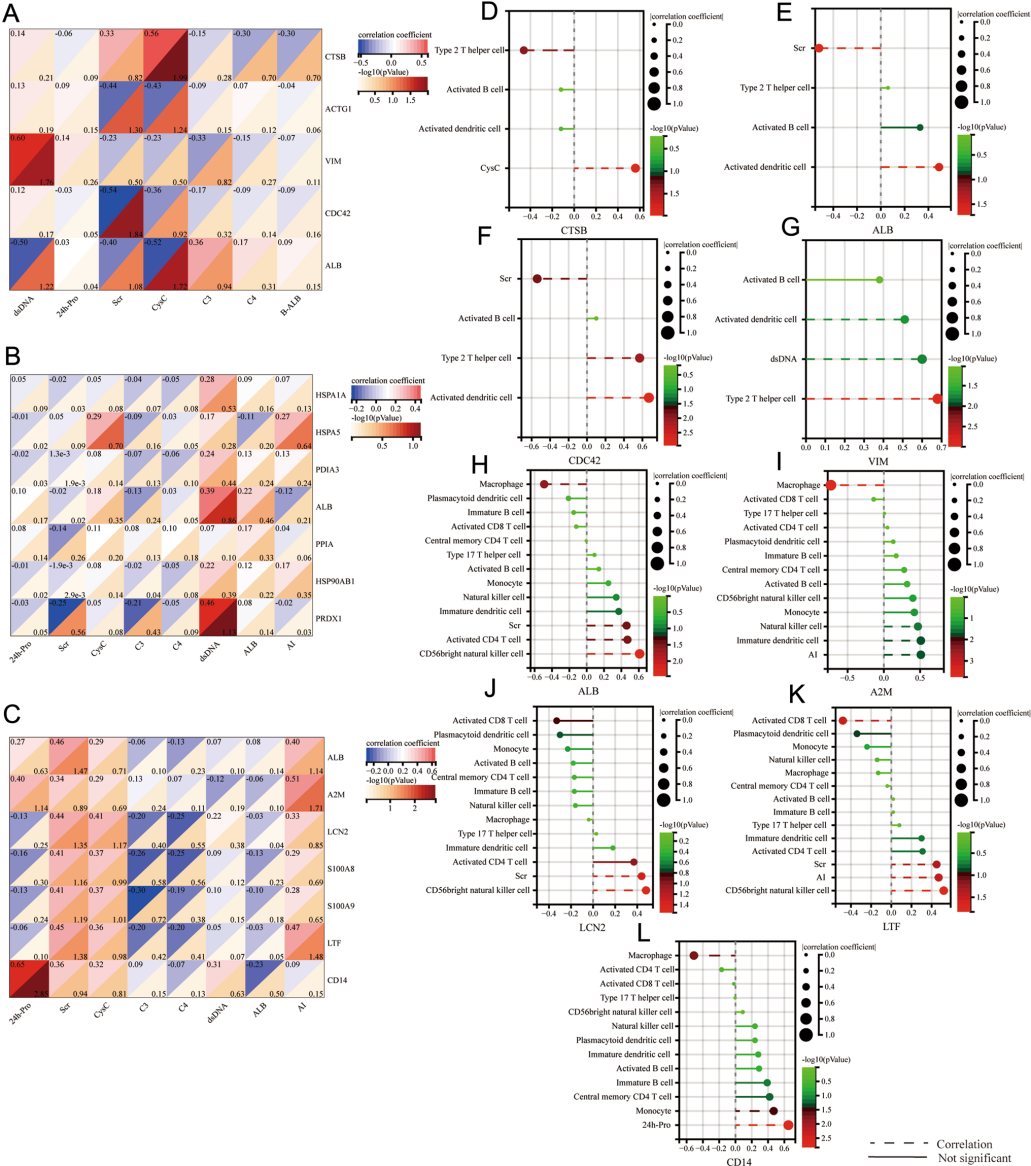
**A**–**C** Correlation between significantly related hub genes and clinical index in renal glomerulus, interstitial, and tubules of LN. **D**–**L** The lollipop graph shows the 9 proteins and the correlation with clinical index and immune enrichment score of differentially immune cells between the LN and NC groups

To further evaluate the correlations between the proteins that these hub genes represent and clinical indices, Spearman’s correlation analysis was performed as shown in Fig. 7. In the glomerulus, we found that cystatin C was significantly correlated with ALB and CTSB but negatively correlated with ALB and positively correlated with CTSB (Fig. 7A). CTSB was negatively correlated with the Type 2 helper T cells based on immune score (Fig. 7D). CDC42 decreased in the sample of LN group and negatively related to the Scr, which shows that it may be a protective protein. CDC42 and VIM were positively correlated with activated dendritic cells and Type 2 helper T cells (Fig. 7F, G). ALB, LCN2, and LTF are associated with Scr in renal tubules, and A2M and LTF are associated with the AI index. LCN2 and LTF positively correlate with the immune scores of CD56 bright natural killer cells (Fig. 7J, K). The immune score of A2M was positively correlated with immature dendritic and natural killer cells (Fig. 7I). CD14 was positively correlated with 24-h urine protein and monocyte (Fig. 7L). The clinical relationship of these genes suggested their potential as biomarkers representative of different renal sites and may be associated with mechanisms of disease progression.

In addition, we found six secreted proteins among the nine key immune-related proteins above based on the Human Protein Atlas (HPA) database (Table 2). By searching the Urinary Protein Biomarker Database (UPBD), these proteins increased in some kidney diseases, which means they have potential in diagnosis and prognosis as candidate biomarkers.

**Table 2:** Potential candidate key proteins

Protein	Secreted protein (HAP)	Secreted to blood	Detected in urine (UPBD)	Increased in kidney diseases (UPBD)
CTSB	Y	Y	Y	IgA glomerulonephritis
VIM	N	/	/	
CDC42	N	/	/	
ALB	Y	Y	Y	IgA glomerulonephritis, Acute kidney failure, Diabetic Nephropathy, Nephrotic syndrome
A2M	Y	Y	Y	Nephrotic syndrome
LCN2	Y	Y	Y	Acute kidney failure, Renal fibrosis, Nephrotic syndrome, Diabetic Nephropathy, Nephrotic syndrome with diffuse membranous glomerulonephritis, Congenital hydronephrosis, Kidney transplant rejection, Contrast-induced Nephropathy, Lupus Glomerulonephritis, Autosomal Dominant Polycystic Kidney Disease
LTF	Y	Y	Y	Congenital occlusion of ureteropelvic junction
CD14	Y	Y	Y	Kidney transplant rejection

*Y* yes, *N* no, *HPA* The Human Protein Atlas database, *UPBD* Urinary Protein Biomarker database

### Immune infiltration characteristic between three compartments

The pathophysiological alteration of the renal regions contributed significantly to the development of the disease. The fractions and enrichment scores of immune infiltrate cells were significantly different among glomerulus, interstitial, and tubules such as native B cells (Fig. S6). Interestingly, in the control group, the degree of difference between the proteins in each compartment of the kidney was more abundant and conspicuous (Fig. S6A, B). For example, in normal kidneys, there are differences in immature dendritic cells between glomeruli and interstitial and between interstitial and tubules. However, this type of cell was not significantly different between each compartment in the LN group (*P* > 0.05), suggesting that the occurrence of LN may alter the abundance of cell types between kidney regions. A significant increase in the subpopulation of the neutrophils and native B cells marked the samples of the glomerulus. In contrast, the tubule samples were characterized by the remarkably highest quiescent immune cells, such as resting NK cells and resting memory CD4 T cells (Fig. S6C, D). The abundance of active infiltrating lymphocyte was significantly associated with antigen–antibody interactions, suggesting that the glomerulus was a much more important region for immune response while renal tubule was characterized by innate immune cell infiltration.

**Fig. 8:**
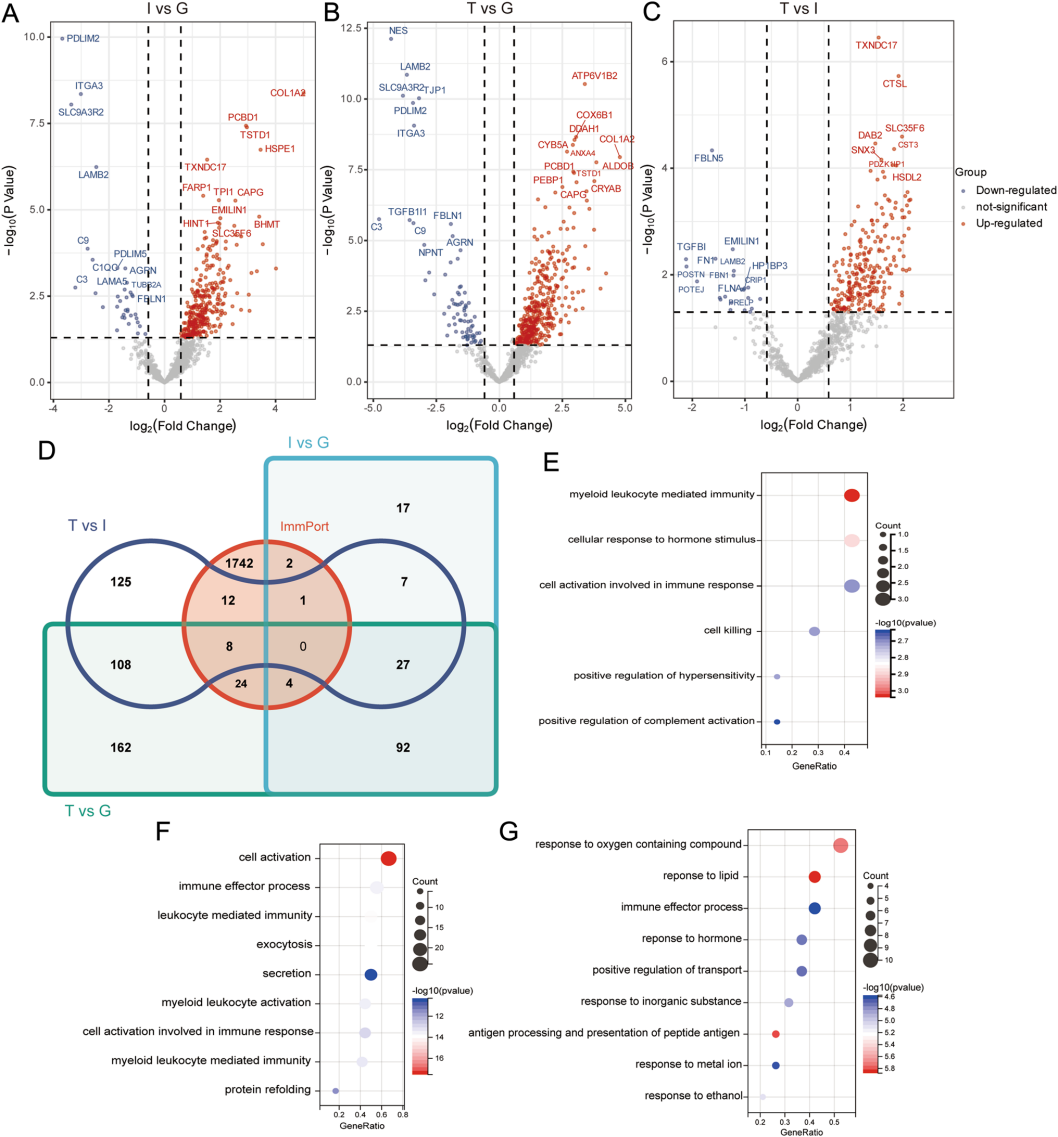
The volcano plots show DEPs between different kidney compartments in LN, **A** I vs G: interstitial versus glomerulus, **B** T vs G: tubules versus glomerulus, **C** T vs I: tubules versus interstitial. **D** Immune-related gene from ImmPort data sources versus DEPs between three compartments Venn diagram. GO enrichment analysis of immune-related DEPs between different kidney compartments in LN **E** glomerulus, **F** interstitial, **G** tubules

Subsequently, the DEPs between glomerular, interstitial, and tubules were identified to be further analyzed (Fig. 8A–C). To further evaluate these DEPs between three compartments, immune-related genes were filtrated by comparing them with immune-related genes from the ImmPort dataset. Venn diagrams revealed 7, 36, and 21 immune-related shared genes in the group of interstitial (I) vs glomerulus (G), (Tubules) T vs G, and T vs I, respectively (Fig. 8D). The GO-biological process analysis shows different immune-related terms among three regions. The DEGs between interstitial and glomerulus were significantly enriched in the immune-related biological processes and myeloid leukocyte-mediated immunity and cellular response to hormone stimulus (Fig. 8E). The DEGs between tubules and glomerulus were significantly enriched in the terms: cell activation and leukocyte-mediated immunity (Fig. 8F). While the DEGs between tubules and interstitial were enriched in the terms: response to lipid, antigen processing and presentation of peptide antigen, and response to oxygen-containing compound (Fig. 8G). These results were consistent with previous results that showed more immune-related pathways in the glomerulus.

**Fig. 9:**
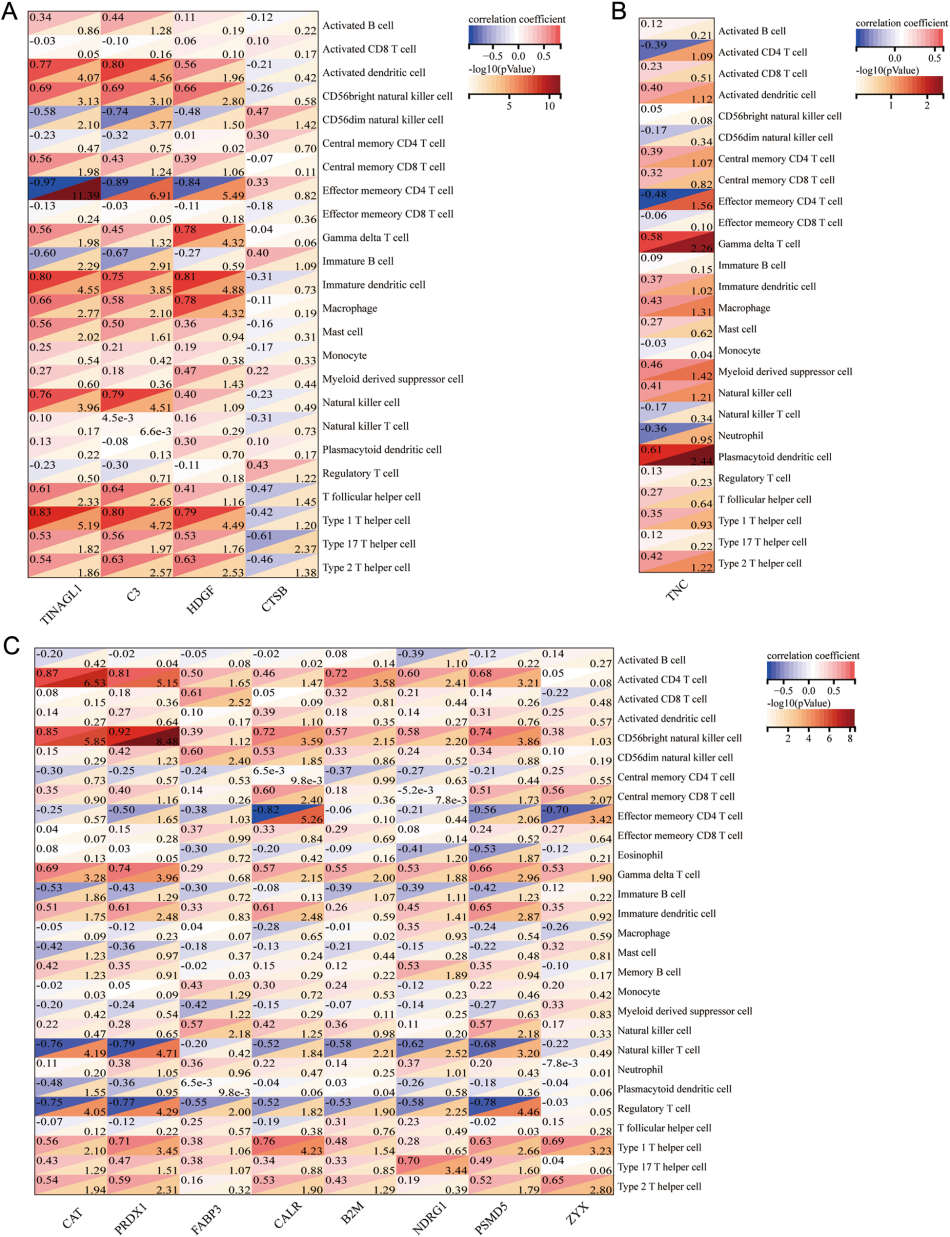
Correlation analysis between significantly dysregulated proteins in three compartments and immune enrichment score in LN, **A** interstitial versus glomerulus, **B** tubules versus glomerulus, **C** tubules versus interstitial

To better characterize the immune profile of each region, differences in the expression patterns of immune genes uniquely expressed in each region were explored. We screened four genes (TINAGL1, C3, HDGF, CTSB) that were only elevated in the glomerulus, one gene (TNC) only elevated in renal interstitial, seven genes (CAT, PRDX1, FABP3, CALR, B2M, NDRG1, PSMD5) only upregulated, and one gene (ZYX) only downregulated in tubules compared to other regions respectively. To explore the potential role of these immune-related differential expressed genes, the correlation of the expression level of these genes with the ICI was presented, as shown in the Fig. 9.

## Discussion

Previous studies on renal proteomics and immune infiltration were often based on bulk tissues, but these intact tissues could not tell which cell populations contributed most to the different compartments of the kidney. Different compartments of kidneys with LN might contain unique subsets of immune cells or cells with activation states that have not previously been described. In this study, we provide detailed proteogenomic profiles and immune infiltration landscapes of different kidney regions in LN patients and control groups.

It revealed the different infiltration of immune cells between the NC and LN groups and the distribution of immune cells between different kidney regions. Through biological function analysis, the immune role of immune-related genes in three regions of the kidney in the pathogenesis of LN was demonstrated. Using immune cell and gene correlation analysis and cyto-hub algorithms, we have identified key immune-related genes that may play an important role in the pathogenesis or serve as biomarkers for diagnosis or disease progression. We screened out ALB, CTSB, LCN2, A2M, CDC42, VIM, LTF, and CD14 to show higher performance in the disease progression of LN.

Finally, we compared the landscape of immune cells and genes between three regions. Comprehensive proteogenomic data in different kidney regions provided a more profound exposition of established LN biology, often with potential therapeutic significance. Proteins, rather than genes, are the ultimate agents of biological function. LN is a typical immune disorder and we comprehensively analyzed it from the perspective of immune infiltration.

B cells were activated and infiltrated significantly in the glomeruli of LN renal tissue, but we also found elevated levels in the interstitial and tubules. The systemic defects of B cell tolerance in SLE lead to the production of high titer autoantibodies, and B cell function in LN is also hyperactive. Development of tertiary lymphoid organs in the tubulointerstitial tissue leads to clonal expansion of B cells, plasma cells, and persistent somatic hypermutation that can lead to intrarenal autoantibody production, inflammation, and tissue injury in proliferative lupus nephritis. B cells play a critical role in the pathogenesis of SLE [16], so B cell depletion therapy remains an attractive treatment option. For instance, Rituximab and Obinutuzumab, the anti-CD20 antibody, can deplete the autoreactive B cells, thereby attenuating the production of autoantibodies involved in disease manifestations [17, 18].

The importance of T cells to LN disease pathology was demonstrated in MRL-lpr mice, which developed less severe kidney disease after T cell depletion [19]. It has been shown that CD8 + T lymphocytes are the primary infiltrating immune cell type in the LN kidney [20]. Tubulointerstitial CD8 + T cell infiltrates correlate with clinical and pathological injury indicators, such as SLEDAI sore, SCr level, proteinuria, the ratio of glomerulosclerosis, and tubulointerstitial inflammation and fibrosis in LN. Tubulointerstitial CD8 + T cells > 130/mm^2^ were independently correlated with an ESRD [21]. CD8 + T also infiltrates glomeruli. Furthermore, urinary CD8 + T cell counts discriminate between active and inactive lupus nephritis more accurately [22].

Autoimmune diseases are associated with dysfunctional regulatory T cells (Tregs) due to their impaired ability to inhibit the function of activated T cell effects and B cells. In SLE patients and lupus-prone mice, reduced Treg cells were positively correlated with disease activity and impaired T cells’ ability to produce IL-2 [23, 24]. There is evidence that the number of naturally occurring Treg cells in the peripheral blood of SLE patients is significantly decreased and dysfunctional compared with normal people. A similar result has been observed in the peripheral blood of LN patients [25, 26]. Some scholars have studied lupus mice and found that the number of CD4 + CD25 + Foxp3 + Treg cells decreased in the advanced stage of the disease [27], suggesting that the decrease in the number of cells is related to the pathogenesis of lupus mice. In vitro Tregs can prevent glomerulonephritis and alleviate LN in lupus mice [28].

Our study showed that Cathepsin B was involved in antigen processing and presentation of the KEGG pathway, which was encoded by the CTSB gene. Mendelian randomization analysis showed a causal relationship between CTSB levels and SLE [29], suggesting it has the potential as a diagnostic/prognostic biomarker. However, the role of CTSB in the kidney of SLE patients is unknown. In this study, it was elevated in the glomeruli, so whether it plays a role in the course of LN disease needs further experimental verification. Microarray analysis of renal macrophages in NZB/W mice (https://www.ncbi.nlm.nih.gov/geo/) suggested that CTSB in the lupus nephritis stage was higher than that in the pre-lupus nephritis stage, but decreased after remission, suggesting that CTSB is involved in the damage of lupus kidney tissue (DataSet: GDS4194).

In addition, our results showed that LCN2 was positively correlated with the immune scores of Natural killer cells. Sun Lingyun’s team [30] demonstrated that the expression levels of PBMC in peripheral blood and LCN2 in kidney tissue were significantly increased in LN patients and significantly positively correlated with acute activity index (AI), chronic activity index (CI), and tubulointerstitial inflammation index of a kidney. Further analysis showed that the expression of LCN2 in renal T cells, macrophages, neutrophils, and renal tubular epithelial cells was significantly increased compared with the control group. In addition, this study provides evidence that LCN2 promotes Th1 cell differentiation through the IL-12/STAT4 pathway in an autocrine or paracrine manner that aggravates LN inflammation. The high expression of LCN2 is significantly correlated with the clinical condition and pathological kidney damage in patients with LN, indicating that LCN2 is involved in the occurrence and development of LN and can be regarded as a potential biomarker. These proteins might potentially develop as diagnostic/prognostic biomarkers for LN.

There are some limitations in this study. One is that the cell model or animal model should test the function of the hub gene to obtain a certain role of the hub gene in lupus nephritis. Furthermore, the gender of the control group is all male, which may be caused by accidental factors. Most LN patients are female, which falls into the morbidity of LN between females and males.

In conclusion, we revealed spatial proteomics and immune signature of LN kidney regions using LCM and DIA, which helped us better understand the functions of different kidney regions contributing to the pathogenesis of LN.

## Supplementary Information

Below is the link to the electronic supplementary material.

Supplementary file1 (DOCX 3904 KB)

Supplementary file2 (DOCX 22 KB)

## Data Availability

The data presented in the study are deposited in the CNGBdb repository, the accession number is CNP0004014.

## References

[1] Jorge A, Wallace ZS, Zhang Y, Lu N, Costenbader KH, Choi HK (2019). All-cause and cause-specific mortality trends of end-stage renal disease due to lupus nephritis from 1995 to 2014. Arthritis Rheumatol.

[2] Parikh SV, Almaani S, Brodsky S, Rovin BH (2020). Update on lupus nephritis: core curriculum 2020. Am J Kidney Dis.

[3] Anders HJ, Saxena R, Zhao MH, Parodis I, Salmon JE, Mohan C (2020). Lupus nephritis. Nat Rev Dis Primers.

[4] Arazi A, Rao DA, Berthier CC, Davidson A, Liu Y, Hoover PJ (2019). The immune cell landscape in kidneys of patients with lupus nephritis. Nat Immunol.

[5] Rovin BH, Parikh SV (2014). Lupus nephritis: the evolving role of novel therapeutics. Am J Kidney Dis.

[6] Weening JJ, D'Agati VD, Schwartz MM, Seshan SV, Alpers CE, Appel GB (2004). The classification of glomerulonephritis in systemic lupus erythematosus revisited. Kidney Int.

[7] Teh CL, Phui VE, Ling GR, Ngu LS, Wan SA, Tan CH (2018). Causes and predictors of mortality in biopsy-proven lupus nephritis: the Sarawak experience. Clin Kidney J.

[8] Leatherwood C, Speyer CB, Feldman CH, D'Silva K, Gómez-Puerta JA, Hoover PJ (2019). Clinical characteristics and renal prognosis associated with interstitial fibrosis and tubular atrophy (IFTA) and vascular injury in lupus nephritis biopsies. Semin Arthritis Rheum.

[9] Hsieh C, Chang A, Brandt D, Guttikonda R, Utset TO, Clark MR (2011). Predicting outcomes of lupus nephritis with tubulointerstitial inflammation and scarring. Arthritis Care Res (Hoboken).

[10] Ko K, Wang J, Perper S, Jiang Y, Yanez D, Kaverina N (2016). Bcl-2 as a therapeutic target in human tubulointerstitial inflammation. Arthritis Rheumatol.

[11] Rao DA, Arazi A, Wofsy D, Diamond B (2020). Design and application of single-cell RNA sequencing to study kidney immune cells in lupus nephritis. Nat Rev Nephrol.

[12] Fava A, Rao DA, Mohan C, Zhang T, Rosenberg A, Fenaroli P (2022). Urine proteomics and renal single-cell transcriptomics implicate interleukin-16 in lupus nephritis. Arthritis Rheumatol.

[13] Austin HA, Muenz LR, Joyce KM, Antonovych TT, Balow JE (1984). Diffuse proliferative lupus nephritis: identification of specific pathologic features affecting renal outcome. Kidney Int.

[14] Charoentong P, Finotello F, Angelova M, Mayer C, Efremova M, Rieder D (2017). Pan-cancer immunogenomic analyses reveal genotype-immunophenotype relationships and predictors of response to checkpoint blockade. Cell Rep.

[15] Newman AM, Liu CL, Green MR, Gentles AJ, Feng W, Xu Y (2015). Robust enumeration of cell subsets from tissue expression profiles. Nat Methods.

[16] Dörner T, Giesecke C, Lipsky PE (2011). Mechanisms of B cell autoimmunity in SLE. Arthritis Res Ther.

[17] Atisha-Fregoso Y, Malkiel S, Harris KM, Byron M, Ding L, Kanaparthi S (2021). Phase II randomized trial of rituximab plus cyclophosphamide followed by belimumab for the treatment of lupus nephritis. Arthritis Rheumatol.

[18] Furie RA, Aroca G, Cascino MD, Garg JP, Rovin BH, Alvarez A (2022). B-cell depletion with obinutuzumab for the treatment of proliferative lupus nephritis: a randomised, double-blind, placebo-controlled trial. Ann Rheum Dis.

[19] Wofsy D, Ledbetter JA, Hendler PL, Seaman WE (1985). Treatment of murine lupus with monoclonal anti-T cell antibody. J Immunol.

[20] Couzi L, Merville P, Deminière C, Moreau JF, Combe C, Pellegrin JL (2007). Predominance of CD8+ T lymphocytes among periglomerular infiltrating cells and link to the prognosis of class III and class IV lupus nephritis. Arthritis Rheum.

[21] Zhang T, Wang M, Zhang J, Feng X, Liu Z, Cheng Z (2021). Association between tubulointerstitial CD8+T cells and renal prognosis in lupus nephritis. Int Immunopharmacol.

[22] Dolff S, Abdulahad WH, Arends S, van Dijk MC, Limburg PC, Kallenberg CG (2013). Urinary CD8+ T-cell counts discriminate between active and inactive lupus nephritis. Arthritis Res Ther.

[23] von Spee-Mayer C, Siegert E, Abdirama D, Rose A, Klaus A, Alexander T (2016). Low-dose interleukin-2 selectively corrects regulatory T cell defects in patients with systemic lupus erythematosus. Ann Rheum Dis.

[24] Venkatadri R, Sabapathy V, Dogan M, Sharma R (2021). Targeting regulatory T cells for therapy of lupus nephritis. Front Pharmacol.

[25] Buckner JH (2010). Mechanisms of impaired regulation by CD4(+)CD25(+)FOXP3(+) regulatory T cells in human autoimmune diseases. Nat Rev Immunol.

[26] Parietti V, Monneaux F, Décossas M, Muller S (2008). Function of CD4+, CD25+ Treg cells in MRL/lpr mice is compromised by intrinsic defects in antigen-presenting cells and effector T cells. Arthritis Rheum.

[27] Gong L, Wang Y, Zhou L, Bai X, Wu S, Zhu F (2014). Activation of toll-like receptor-7 exacerbates lupus nephritis by modulating regulatory T cells. Am J Nephrol.

[28] Scalapino KJ, Tang Q, Bluestone JA, Bonyhadi ML, Daikh DI (2006). Suppression of disease in New Zealand Black/New Zealand White lupus-prone mice by adoptive transfer of ex vivo expanded regulatory T cells. J Immunol.

[29] Mo X, Guo Y, Qian Q, Fu M, Lei S, Zhang Y (2020). Mendelian randomization analysis revealed potential causal factors for systemic lupus erythematosus. Immunology.

[30] Chen W, Li W, Zhang Z, Tang X, Wu S, Yao G (2020). Lipocalin-2 exacerbates lupus nephritis by promoting Th1 cell differentiation. J Am Soc Nephrol.

