# Supplementary material for: Spatial proteomics landscape and immune signature analysis of renal sample of lupus nephritis based on laser-captured microsection
Source: Inflamm Res. 2023 Jul 20;72(8):1603–20. doi: 10.1007/s00011-023-01767-3 (PMC10499763; doi:10.1007/s00011-023-01767-3)
Supplement: Supplementary file 1 — Supplementary file1 (DOCX 3904 KB) [file 11_2023_1767_MOESM1_ESM.docx]

**Spatial proteomics landscape and immune signature analysis of renal sample of lupus nephritis based on lacer captured microsection**

Fengping Zheng^1,2*^, Donge Tang^2*^, Shanshan Li^2^, Zhifeng Luo3, Yueqi Song^2^, Yinxin Huang ^2^, Qing Gan^2^, Hanyong Liu^2^, Xinzhou Zhang^2^, Dongzhou Liu^2^, Qingwen Wang4, Zuying Xiong^1^, Yong Dai^2,5^


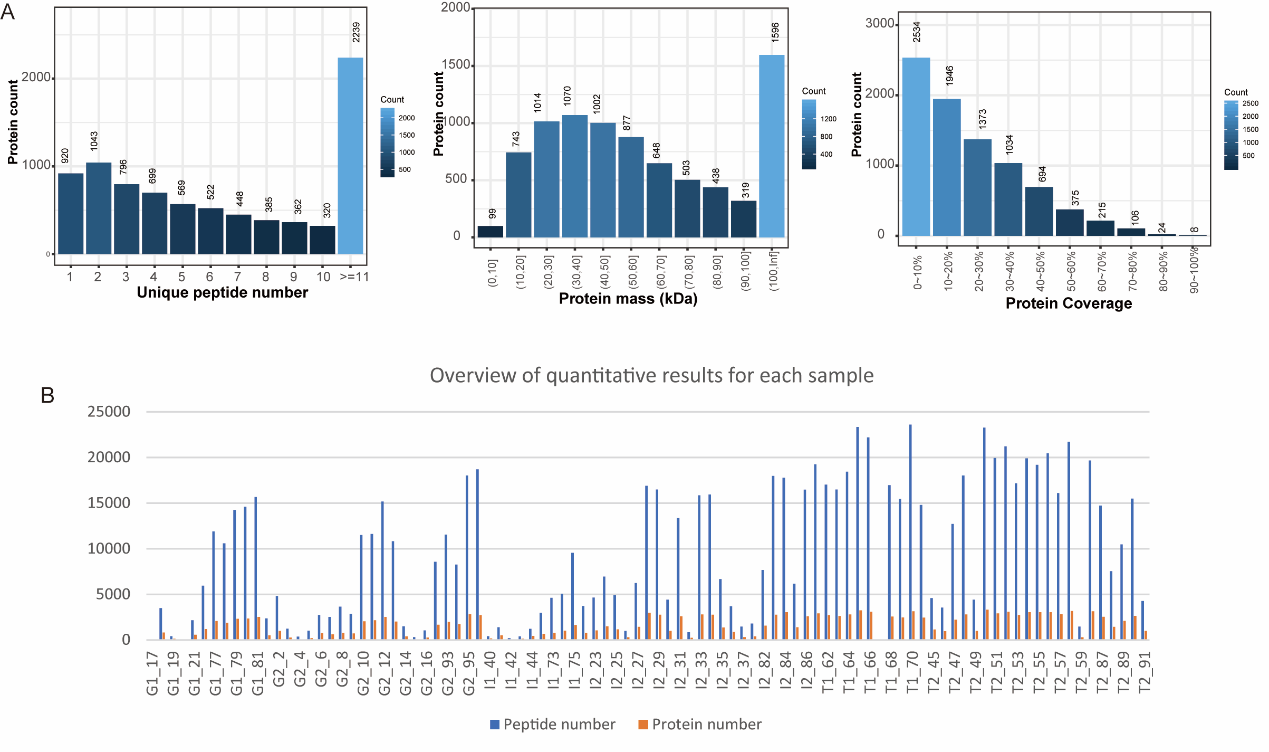


Figure S1 Basic statistics of the DDA identification results(A) and DIA quantitative statistics of each sample(B).


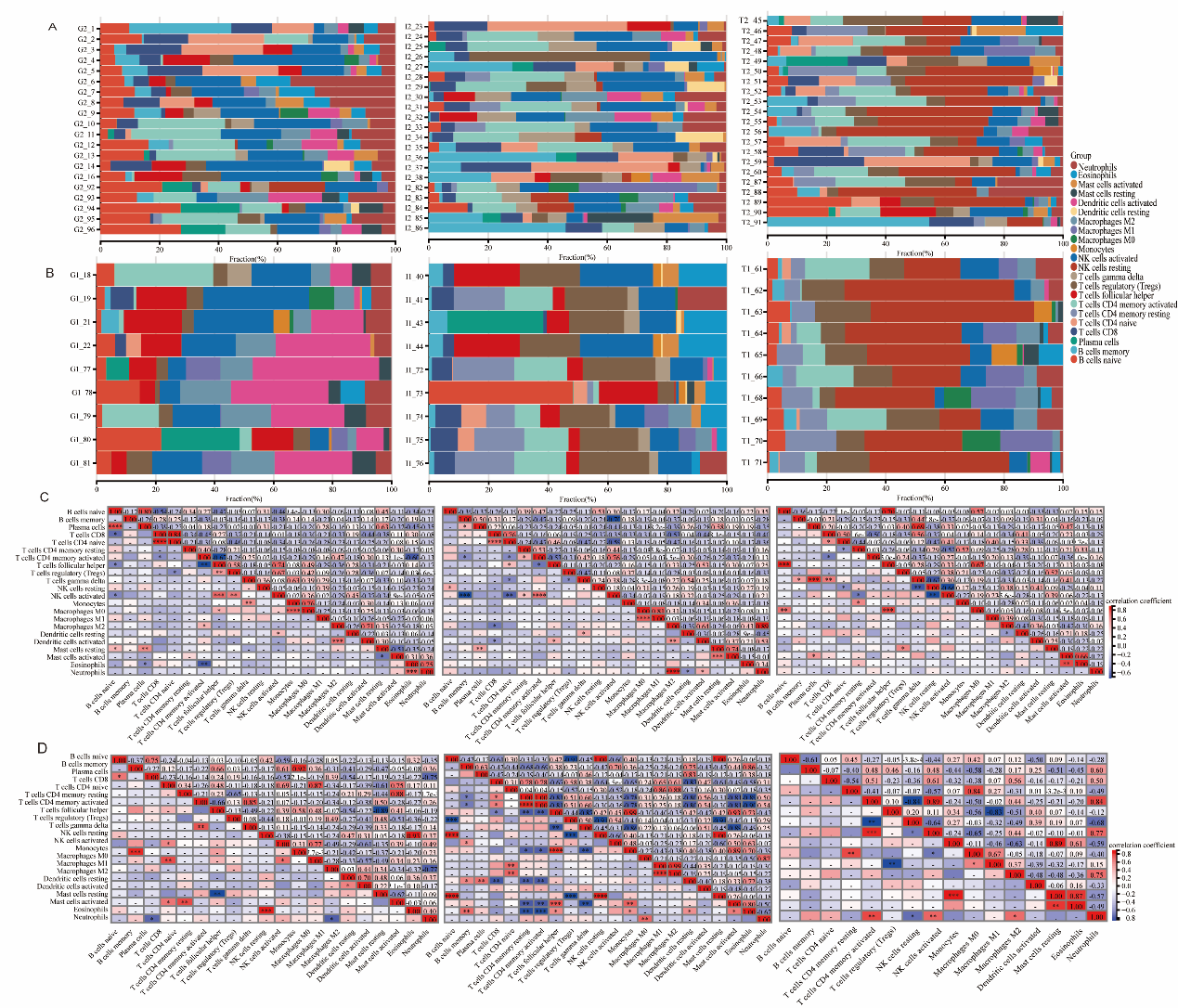


Fig. S2 Immune characteristics between LN and NC group. (A) Immune cell content stacking plot of glomerulus, interstitial and tubules of LN patients, respectively. (B) Immune cell content stacking plot of glomerulus, interstitial and tubules of normal kidney tissues, respectively. (C) Correlations between 22 immune cells in LN group. (D) Correlations between 22 immune cells in NC group. Red: positive correlation; blue: negative correlation.


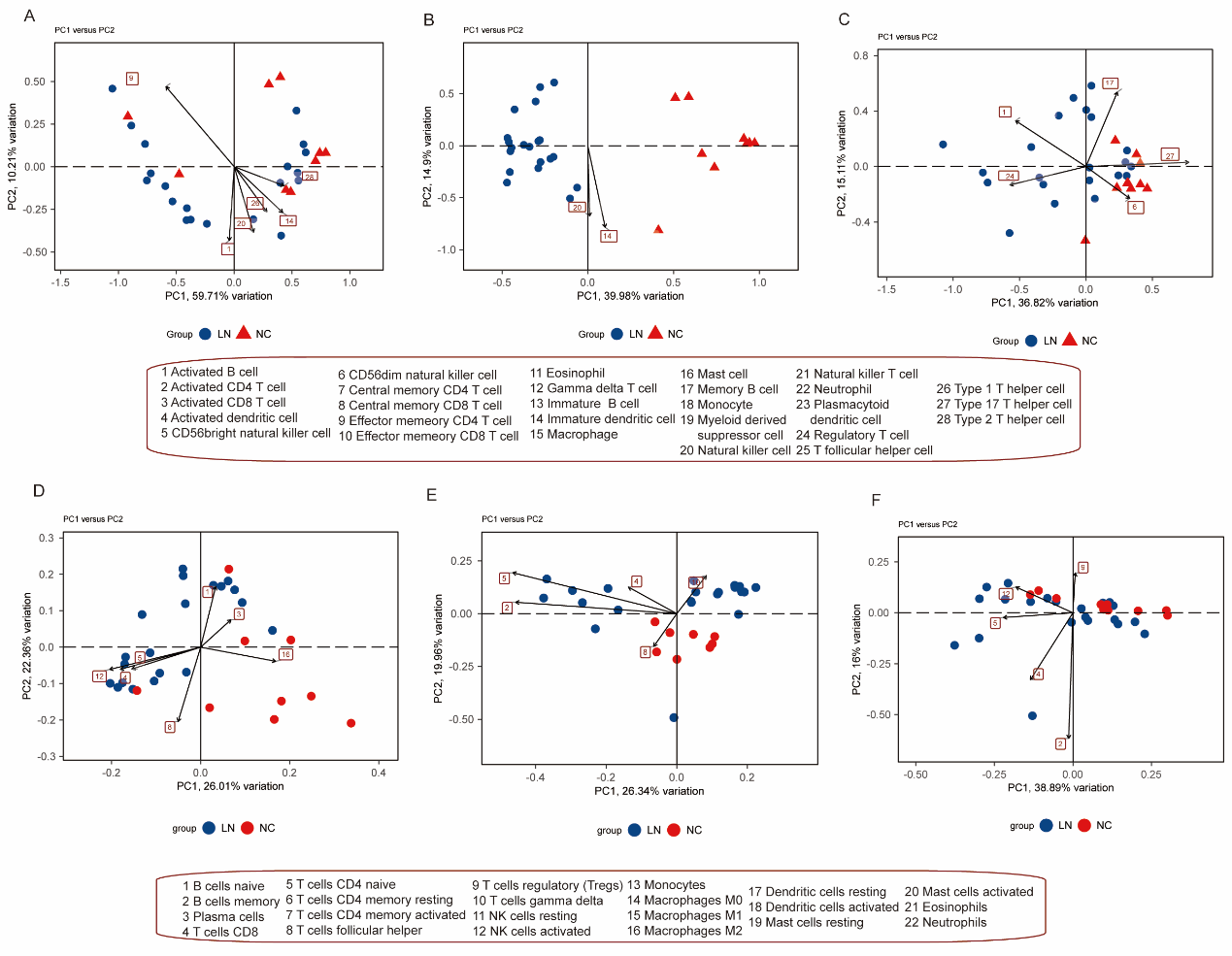


Fig. S3 PCA bi-plot analysis based on immune characteristics in (A) glomerulus, (B) interstitial and (C) tubules and based on immune enrichment score in (D) glomerulus, (E) interstitial and (F) tubules.


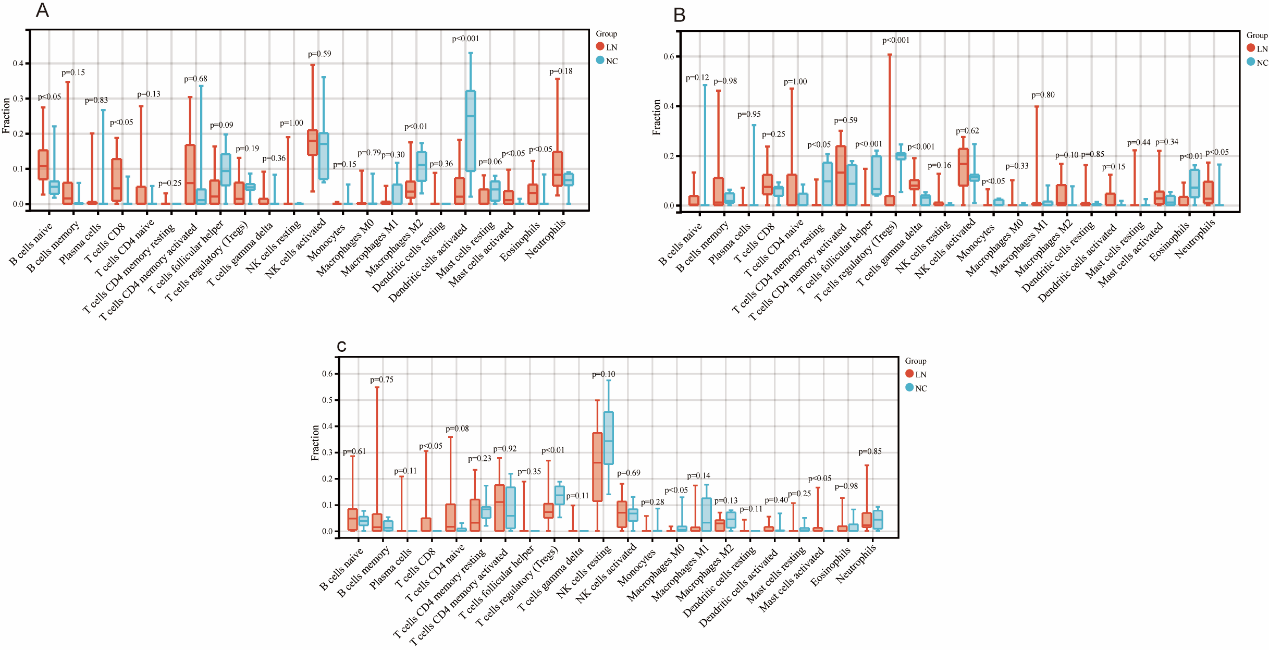


Fig. S4 Box plot of Immune cell proportion between LN and NC group in (A) glomerulus (B) interstitial (C) tubules.


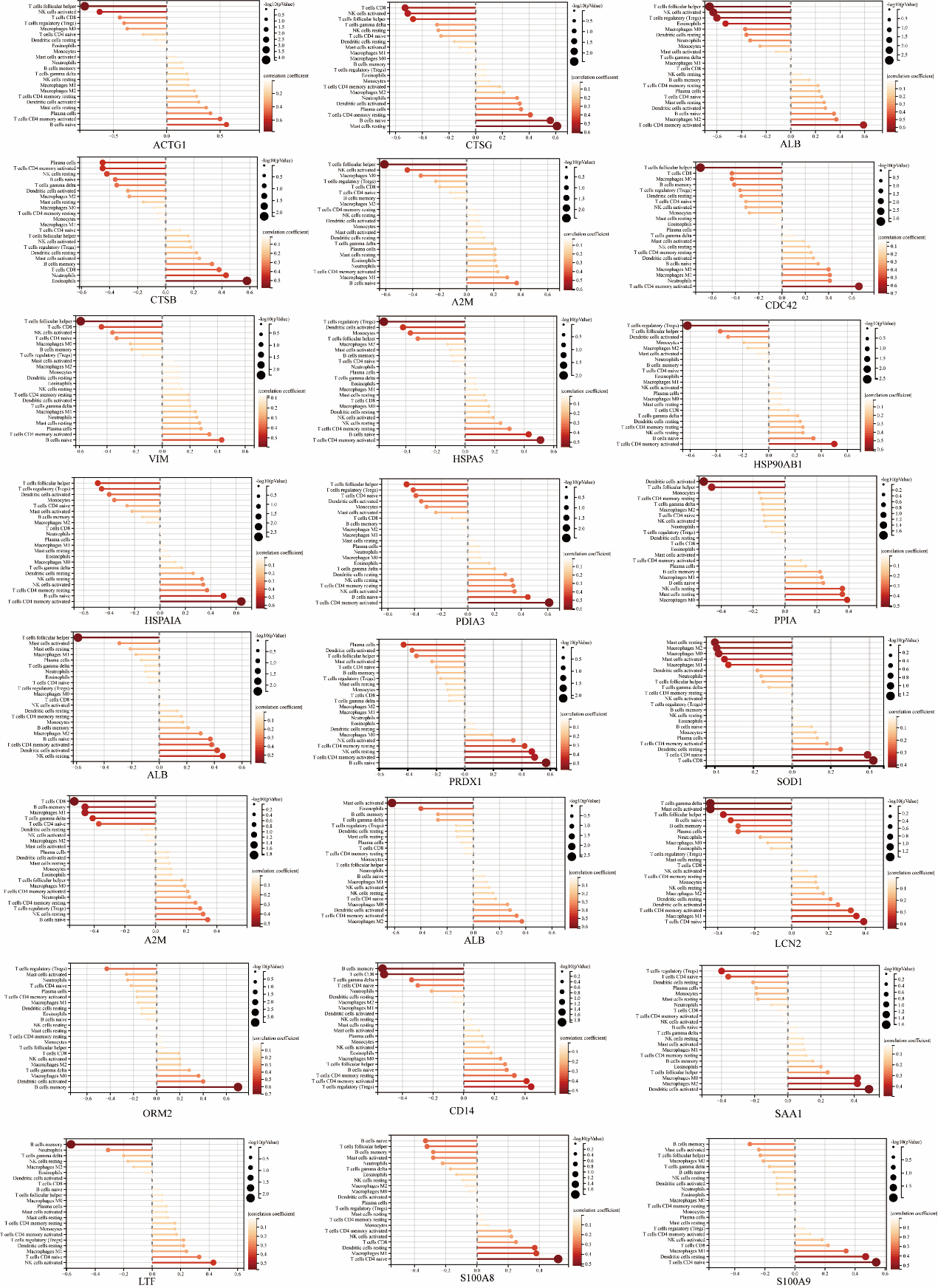


Fig. S5 Correlation analysis between hub genes and immune infiltration cells in LN.


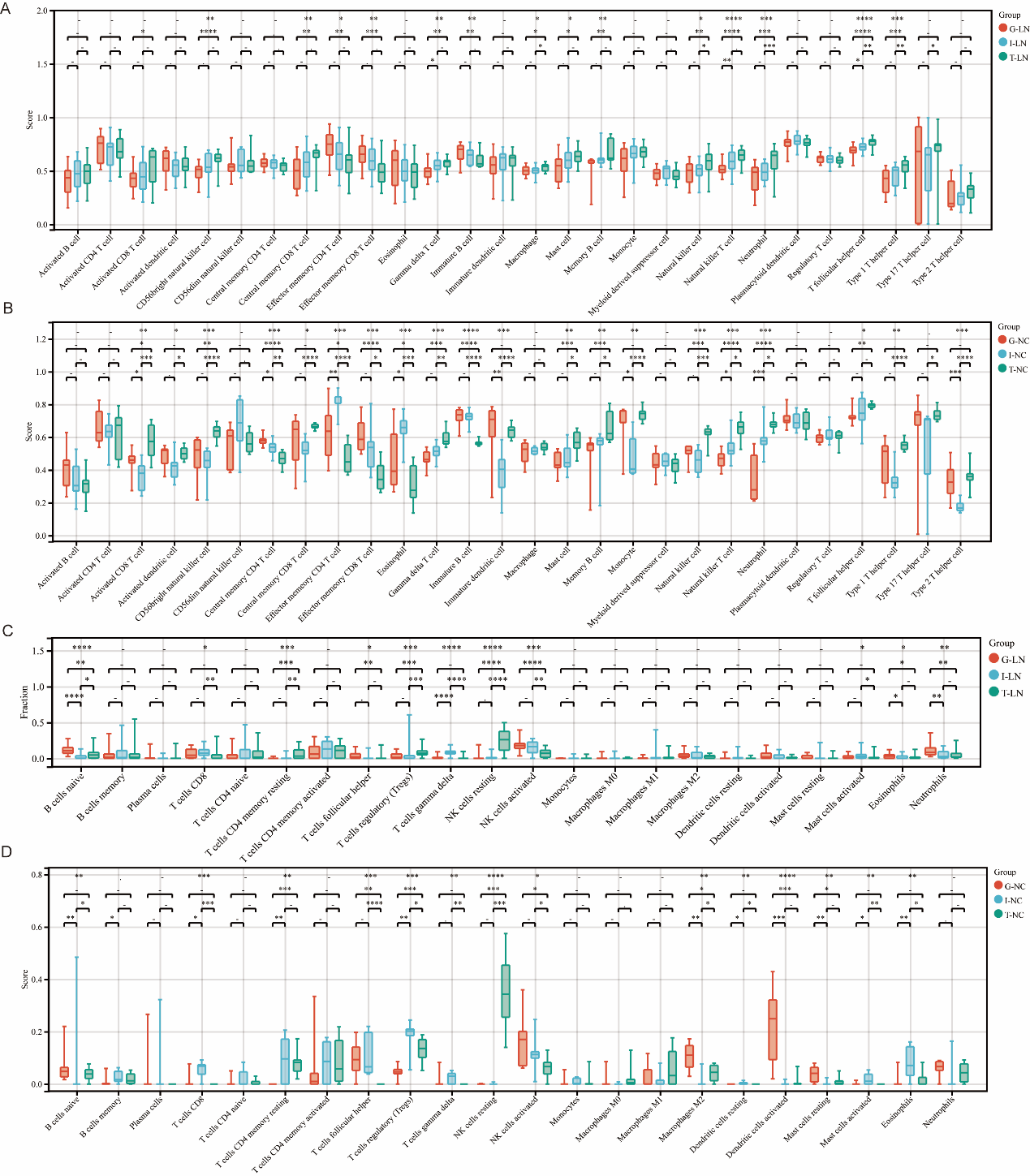


Fig S6 Box plot of fractions of immune infiltrate cells in between three compartments in (A)LN and (B)NC group; Box plot of the immune enrichment score between three compartments in (C)LN and (D)NC group.
