# Supplementary material for: Spatial proteomics landscape and immune signature analysis of renal sample of lupus nephritis based on laser-captured microsection
Source: Inflamm Res. 2023 Jul 20;72(8):1603–20. doi: 10.1007/s00011-023-01767-3 (PMC10499763; doi:10.1007/s00011-023-01767-3)
Supplement: Supplementary file 2 — Supplementary file2 (DOCX 22 KB) [file 11_2023_1767_MOESM2_ESM.docx]

Table S1 Top 20 proteins differentially expressed in three subareas

| Protein | Description | log2FC | *P*-value |
| --- | --- | --- | --- |
| glomerulus | | | |
| P01619 | Immunoglobulin kappa variable 3-20 | 4.663787334 | 0.00045178 |
| P0DOX5 | Immunoglobulin gamma-1 heavy chain | 4.518889408 | 1.60E-07 |
| P01860 | Immunoglobulin heavy constant gamma 3 | 4.465777327 | 9.48E-06 |
| O60656 | UDP-glucuronosyltransferase 1-9 | 4.461642196 | 0.00088732 |
| Q9UG63 | ATP-binding cassette sub-family F member 2 | 4.424300333 | 0.00013836 |
| P02747 | Complement C1q subcomponent subunit C | 4.218407556 | 0.00077297 |
| P08779 | Keratin, type I cytoskeletal 16 | 4.147645442 | 0.00189858 |
| P16662 | UDP-glucuronosyltransferase 2B7 | 4.016893305 | 0.00659355 |
| P08727 | Keratin, type I cytoskeletal 19 | 3.926775129 | 9.43E-05 |
| P02766 | Transthyretin | 3.8692915 | 0.00561463 |
| O43896 | Kinesin-like protein KIF1C | -5.479248296 | 6.58E-08 |
| Q9BVM2 | Protein DPCD | -4.414085464 | 0.00048538 |
| Q13045 | Protein flightless-1 homolog | -4.144711333 | 0.00018545 |
| P30153 | Serine/threonine-protein phosphatase 2A 65 kDa regulatory subunit A alpha isoform | -3.805800381 | 2.67E-06 |
| P20774 | Mimecan | -3.686032691 | 0.00601635 |
| Q8N0U8 | Vitamin K epoxide reductase complex subunit 1-like protein 1 | -3.658154835 | 1.87E-05 |
| A5YKK6 | CCR4-NOT transcription complex subunit 1 | -3.545488392 | 1.82E-06 |
| Q9P260 | RAB11-binding protein RELCH | -3.537686301 | 2.46E-07 |
| Q2TAA2 | Isoamyl acetate-hydrolyzing esterase 1 homolog | -3.506403704 | 1.85E-06 |
| P12270 | Nucleoprotein TPR | -3.448194441 | 4.79E-06 |
| Interstitium | | | |
| P48444 | Coatomer subunit delta | 5.228980093 | 2.79E-05 |
| Q96JB2 | Conserved oligomeric Golgi complex subunit 3 | 4.740328557 | 0.00039804 |
| Q9H2M9 | Rab3 GTPase-activating protein non-catalytic subunit | 4.658873984 | 0.02161906 |
| P46782 | 40S ribosomal protein S5 | 4.529936509 | 2.56E-05 |
| P0DOX5 | Immunoglobulin gamma-1 heavy chain | 4.426340068 | 9.76E-07 |
| P07951 | Tropomyosin beta chain | 4.294583031 | 0.00012031 |
| Q86V81 | THO complex subunit 4 | 4.289678672 | 2.33E-09 |
| P69905 | Hemoglobin subunit alpha | 4.178732795 | 4.73E-05 |
| P55060 | Exportin-2 | 4.155715355 | 8.19E-05 |
| P12110 | Collagen alpha-2(VI) chain | 4.064663175 | 1.67E-09 |
| P08603 | Complement factor H | -7.985309296 | 2.30E-07 |
| P02679 | Fibrinogen gamma chain | -7.875065271 | 8.00E-05 |
| P04114 | Apolipoprotein B-100 | -7.518882248 | 1.45E-06 |
| P02790 | Hemopexin | -7.291261992 | 0.00121642 |
| P00450 | Ceruloplasmin | -7.033155327 | 1.57E-06 |
| P43652 | Afamin | -6.869268232 | 0.01567837 |
| P04217 | Alpha-1B-glycoprotein | -6.360499263 | 1.05E-07 |
| P02763 | Alpha-1-acid glycoprotein 1 | -5.886045032 | 0.00924812 |
| P01042 | Kininogen-1 | -5.771249353 | 3.62E-05 |
| P0C0L5 | Complement C4-B | -5.760548595 | 0.01233411 |
| Tubules | | | |
| Q9Y485 | DmX-like protein 1 | 6.528380556 | 0.00276353 |
| P0DOX5 | Immunoglobulin gamma-1 heavy chain | 4.239865801 | 3.23E-08 |
| P01834 | Immunoglobulin kappa constant | 4.142139984 | 5.76E-06 |
| P69892 | Hemoglobin subunit gamma-2 | 3.753567906 | 0.00629942 |
| O60884 | DnaJ homolog subfamily A member 2 | 3.563466191 | 0.00035888 |
| P49913 | Cathelicidin antimicrobial peptide | 3.394762896 | 0.03544706 |
| P23083 | Immunoglobulin heavy variable 1-2 | 3.374773255 | 0.00013244 |
| P01619 | Immunoglobulin kappa variable 3-20 | 3.215450328 | 0.00086843 |
| P01861 | Immunoglobulin heavy constant gamma 4 | 3.097224872 | 0.00627909 |
| P05109 | Protein S100-A8 | 2.890218691 | 0.00660173 |
| Q16512 | Serine/threonine-protein kinase N1 | -3.130121713 | 0.03587691 |
| Q13621 | Solute carrier family 12 member 1 | -2.986905503 | 0.00059324 |
| P0DJI8 | Serum amyloid A-1 protein | -2.894811629 | 0.00315323 |
| P62841 | 40S ribosomal protein S15 | -2.886604522 | 0.02159814 |
| P09210 | Glutathione S-transferase A2 | -2.845432395 | 2.64E-05 |
| Q7Z4W1 | L-xylulose reductase | -2.630177764 | 0.00284839 |
| P0CG12 | Decreased expression in renal and prostate cancer protein | -2.606633907 | 0.01905803 |
| P11277 | Spectrin beta chain, erythrocytic | -2.537822586 | 0.0301247 |
| P36222 | Chitinase-3-like protein 1 | -2.492426802 | 0.0283955 |
| Q92696 | Geranylgeranyl transferase type-2 subunit alpha | -2.323912897 | 0.02860845 |
